# 

**Published:** 1910-08-27

**Authors:** 


### Appointments and Resignations.

Miss Maude Marsden, M.B., Ch.B., has been appointed assistant medical officer at the out-patient department of the Manchester Children's Hospital, Pendlebury, for a period of six months.

Miss Nicholson, of St. Bartholomew's Hospital, has been appointed lady superintendent at the Manchester Children's Hospital at Pendlebury.

### Gifts and Bequests.

Under the will of the late Mr. Samuel S. M. Howkins, of Leicester, proved on the 2nd inst., a legacy of £100 is bequeathed to the Leicester Infirmary, free of duty.

Mr. William Alexander Pattison, of Valley Drive, Harrogate, bequeathed £500 to the Victoria Hospital, Southend, and an additional sum of £800 to the same institution.

Prince Francis of Teck acknowledges £210 from Messrs. Mosenthal, Sons and Co., and £100 from Miss Gladys Duncan, in response to his appeal on behalf of the Middlesex Hospital.

Miss Elizabeth Didsbury, of Eastwood Villas, Rotherham, Yorks, willed £6,000 to the trustees of Rotherham Hospital and Dispensary and £1,000 to the Jessop Hospital for Women, Sheffield.

The Chung Yee Tong (Chinese Sailors' Society) has sent a contribution of £20 to the funds of the Seamen's Hospital Society, Dreadnought, in recognition of treatment afforded to sick and injured Chinese seamen in this country.

The Secretary and Manager to the York County Hospital has received a letter from Mr. H. H. Riley-Smith, J.P., enclosing a cheque for £1,000 from his brother and himself, which has also been the means of raising another £2,000.

Mrs. Ellen Jane Gordon, of Ingleden, St. Michael's, Tenterden, Kent, who died on June 15, widow of Admiral George Gordon, left estate valued at £27,986 gross, with net personalty £22,148. She left £100 each to the West Kent General Hospital, Maidstone, the Royal Eye Hospital, St. George's Circus, S.E., and the Royal National Hospital for Consumption and Diseases of the Chest, Ventnor, Isle of Wight.

The Treasurer of the Leicester Infirmary has received from Mr. W. B. Clark a donation of £25 in memory of his father, the late Mr. J. Webster Clark, a generous benefactor of the Infirmary. A sum of £20 14s. 1d. has also been received as the result of the United Friendly Societies' church parade at Melton Mowbray, and a sum of £15 from the Gillette Safety Razor Co. to the furnishing fund of the Nurses' New Home.

Alderman Thomas William Oakshott, of Liverpool, who died on June 13, aged seventy-six, bequeathed £500 each to the Liverpool Royal Infirmary, the Consumption Hospital, Liverpool, the Woolton Convalescent Institution, the Stanley Hospital, Liverpool, and the Southern Hospital, Liverpool; and £250 each to the Ladies' Charity and Lying-in Hospital, Liverpool; the Hospital for Cancer and Skin Diseases, Liverpool; the Children's Infirmary, Liverpool; the Bluecoat Hospital, Liverpool; the Birkenhead Borough Hospital, and the Children's Hospital, Birkenhead.

Mrs. Proctor Baker has given £10,000 to Winsley Sanatorium in memory of her husband, formerly a prominent member of the Bristol City Council.

Mrs. Maria Crampton, of 8 Second Avenue, Hove, Sussex, who died on June 30 last, wife of Mr. Thomas Hillas Crampton, bequeathed £1,000 to the Sussex County Hospital, to be associated with the name of her late husband, Mr. Lawrence Fort.

Mr. William Albert Maud, of Coleman Street, E.C., and of Airedale Lodge, Prospect Hill, Walthamstow, N.E., wool broker, of the firm of Messrs. M. Maud and Son, who died on July 20, left £100 each to the Leeds General Infirmary, the London Hospital, and the City of London Hospital for Diseases of the Chest, Victoria Park, N.E.

Mr. John Birkmyre, of Broadstone, Port Glasgow, whose death occurred recently, bequeathed the sum of £5,000 towards the endowment fund of Broadstone Jubilee Hospital, Port Glasgow. The hospital was a golden wedding gift of Mr. and Mrs. John Birkmyre to Port Glasgow two years ago, and was erected and equipped at a cost of over £30,000.

Captain Caulfeild French, of 48 Stephen's Green, Dublin, and of Castle Bernard, Kinmitty, King's County, late of the 94th Regiment of Foot, who died on June 7 last, aged seventy years, left £1,050 to the Royal National Hospital for Consumption for Ireland at Newcastle, county Wicklow, for the endowment of three beds, and £500 each to the Adelaide Hospital, Dublin, the Sir Patrick Dun's Hospital, Dublin, and the Royal Hospital for Incurables, Donnybrook, Dublin.

Mr. Peter Hubert Desvignes, M.R.C.S., of Firsdene, Oatlands Drive, Weybridge, who died on July 14, aged seventy-three, left estate valued at £34,231 gross, with net personalty £34,117. He bequeathed £6,000 to Guy's Hospital, "where I was a student in 1853," to found, endow, and maintain four beds and four cots in memory of his late sister Caroline France Desvignes, to be called the "Desvignes Beds or Cots"; £1,000 to Dr. Barnardo's Homes; £500 to King's College, London, for the promotion of research work in the Bacteriological Laboratory; and £500 each to the Weybridge Cottage Hospital and the British Medical Benevolent Fund. Subject to these bequests, he left the residue of his property, which would appear to amount to rather over £8,000, to Guy's Hospital, as to one-sixth for the Bacteriolgical Laboratory, for the promotion of research work only, and as to five-sixths for additional "Desvignes Beds or Cots," any balance to be applied for the general purposes of the hospital.

Messrs. J. and A. Churchill are about to publish three new editions of well-known text-books. These are "Serum Therapy, Bacterial Therapeutics, and Vaccines," by R. Tanner Hewlett; "The Microscopical Examination of Foods and Drugs," by H. G. Greenish; and "Hernia, Its Cause and Treatment," by R. W. Murray.

### JOTTINGS.

Belfast proposes to erect an additional block at the Royal Victoria Hospital as a memorial to King Edward VII.

The Earl of Warwick will open the Alfred Boyd Memorial Sanatorium, at Little Baddow, at 2 p.m., on Monday, September 12 next.

The Alfred Hospital, Melbourne, where the closing of some wards was imminent owing to lack of funds, has received several donations of £50.

Warwickshire proposes to commemorate the reign of King Edward VII. by establishing an open-air hospital for the treatment of consumption.

The Women's Hospital Funds (Melbourne) have received the sum of £5,400 from the Easter appeal, and a large new department and nurses' home, where every nurse has a bedroom to herself, have just been opened.

While answering a call on July 26, an ambulance of the German Hospital, Williamsburg, N.Y., was struck by a trolley car of the Myrtle Avenue line. Both the surgeon and the driver of the ambulance were injured.

The King Edward VII. Memorial Fund for Bath has reached a total of £460. It has not yet been decided what the memorial shall be, but it has been suggested that the money shall be expended in connection with the Royal United Hospital.

The collections on Hospital Day at Rye and sums received since amounted to £105 17s. 2d., and after paying expenses the committee was enabled to remit to the hospital the sum of £98 17s. 6d., which, with other subscriptions, made a grand total of £150, and breaks the record.

The Kent medical officer has placed before the County Council a scheme for establishing sanatoria for the treatment of consumption in different areas of Kent. It is proposed that sanatoria shall be established for each of the following four districts: East Kent, North Kent, West Kent, South and Central Kent. A conference of local medical officers is being summoned to consider the question.

The Medical Officer of Health for Hertfordshire has been directed by the County Council to hold local inquiries at Hitchin, Baldock, and Stevenage into the necessity of isolation hospitals being established there. The present state of affairs is most unsatisfactory, these places being without any means whatever for isolating cases of infectious disease. The County Council has also urged the Hemel Hempstead Hospital Board to immediately carry out, without further delay, the proposed extension of the Isolation Hospital premises. At present the hospital is over-crowded, the accommodation for nurses too small, and it is only safe to admit one kind of infectious disease at a time.

The Coventry Police have in their charge a man who has appropriated a Hospital donation-box in one of the public-houses in this city. He was brought before the magistrates on Friday last, but a remand was asked for to enable the police to make further investigations, as it is known that at least five of these boxes have been stolen from various licensed premises in the city. About two hundred and forty of these boxes were distributed amongst the various licensed houses last Christmas, and in the majority of cases are being well supported. In one house the box was practically filled in about three weeks, and contained over £1 in small coins. The committee of the local Licensed Victuallers' Association has very kindly undertaken the collection of the amounts annually. The first collection is to take place early next month.

Miss Ann Woodward, of Gillott Road, Birmingham, who died on July 3, leaving estate valued at £9,370 gross, with net personalty £9,283, bequeathed £500 each to the Birmingham General Hospital, the Birmingham and Midland Skin and Urinary Hospital, and the Birmingham and Midland Eye Hospital; £200 to the Birmingham General Dispensary, Union Street; and £100 to the Royal Orthopædic and Spinal Hospital, Birmingham.

The following Papers will be read by British contributors at the International Congress on Diseases of Occupation which meets at Brussels on September 10: "The English Workmen's Compensation Act; with Special Reference to Hernia, Lumbago, Heat Stroke, and Caisson Disease," by Dr. D. J. Collie; "Smallpox as an Industrial Accident," by Dr. H. E. Corbin, Medical Officer of Health, Stockport; "The Function of the Certifying Factory Surgeon," by Dr. W. F. Dearden; "Certificates of Fitness in the Cotton Trade," by Dr. A. Glen Park; "Statistical Studies of Phthisis as an Occupational Disease," by Dr. T. D. Lister; "Descriptions of Systems of Medical Service Adopted by Leading British Railway Companies," by Dr. J. G. McBride, Dr. T. M. Watts, and Dr. W. F. Dearden; "Experience of Ankylostomiasis in Cornwall," by Dr. A. E. Boycott; "Incidents in the Life-History of Ankylostoma Duodenale," by Professor Sir Thomas Oliver; "Glass-Workers' Cataract," by Dr. W. Robinson; "Gaseous Poisons; with Special Reference to Hydrogen Arsenide and Hydrogen Phosphide," by Professor John Glaister; "A Contribution to the Study of Aniline Poisoning," by Dr. R. Prosser White, surgeon to the Roburite Explosives Company, and Dr. A. Sellars, Public Health Laboratory, Manchester; "Some Unusual Features of Lead Poisoning," by Professor Sir Thomas Oliver; "Ten Years' Experience of Notification of Lead Poisoning," by Dr. T. M. Legge; "Early Indications of Lead Poisoning," by Dr. S. King Alcock, certifying surgeon, Burslem.

**For Hospitals and Institutions.**

| | |
|---|---|
| Analysis Ledger. 3 vols. (Uniform with the new Index of Classification adopted by the King's Hospital Fund) ... ... ... ... | £1 12s. 6d. |
| Cash Book ... ... ... ... ... ... ... | 12s. 9d. |
| Cash Analysis and Receipt Book ... ... ... | 16s. 6d. |
| Secretary's Petty Cash Book ... ... ... | 13s. 0d. |
| List or Register of Annual Subscribers ... ... | £1 0s. 0d. |
| Linen Register ... ... ... ... ... ... | 7s. 6d. |

And many other Ledgers necessary in the office of large and small institutions.

Of all booksellers or of The Scientific Press, Limited, 28 and 29 Southampton Street, Strand, London, W.C.

